# Role of Vaccine Hesitancy, eHealth Literacy, and Vaccine Literacy in Young Adults’ COVID-19 Vaccine Uptake Intention in a Lower-Middle-Income Country

**DOI:** 10.3390/vaccines9121405

**Published:** 2021-11-29

**Authors:** Rima Nath, Asif Imtiaz, Shobod Deba Nath, Emran Hasan

**Affiliations:** 1 Department of Public Health, North South University, Dhaka 1229, Bangladesh; rima.nath@northsouth.edu; 2 Department of MIS, University of Dhaka, Dhaka 1000, Bangladesh; asifimtiaz.mis@du.ac.bd; 3 Department of Health Policy, The London School of Economics and Political Science (LSE), London WC2A 2AE, UK; 4 Department of International Business, University of Dhaka, Dhaka 1205, Bangladesh; 5 Department of Economics, Bangladesh University of Professionals (BUP), Dhaka 1216, Bangladesh; emran.hasan@bup.edu.bd

**Keywords:** vaccine uptake intention, vaccine hesitancy, eHealth literacy, vaccine literacy, COVID-19

## Abstract

Various control measures, including vaccination, have been taken to flatten the COVID-19 epidemic curve across the globe. However, in Bangladesh, many young adults, considered the asymptomatic transmitter of the disease, are waiting to get their first shot. Therefore, the potential predictors of the young adults’ vaccine uptake intention are significant to ensure their maximum vaccination when available to them. This study examined how vaccine hesitancy, eHealth literacy, and vaccine literacy are associated with young adults’ COVID-19 vaccine uptake intention in a lower-middle-income country. A total of 343 young adults participated in the study. Using ordinary least square and probit estimation, we examined the effect of the explanatory variables of interest on vaccine uptake intention. Vaccine hesitancy emerged as the strongest predictor of vaccine uptake intention. eHealth literacy shared a positive association with vaccine uptake intention, while vaccine literacy had no significant association. To make young adults feel more confident about the vaccine, transmitting the latest vaccine safety updates through authentic channels is essential. The government can aim to enhance the eHealth literacy of young adults as an increased level of eHealth literacy will enable young adults to extract reliable health-related information more efficiently than ever.

## 1. Introduction

The COVID-19 pandemic has overwhelmed the health system in countries ranging from Low- and Middle-Income countries (LMICs) to High-Income countries (HIC) [1,2,3]. During the early stages of the pandemic, lack of immunization and the absence of specific treatment prompted the implementation of non-pharmaceutic interventions, including handwashing, social distancing, and lockdown/movement restrictions to curb the spread of the COVID-19 virus [4,5,6]. However, implementation of these mitigation measures, in turn, impacted the livelihoods of the people, especially the poor, vulnerable, and marginalized people. Given the circumstances, developing an effective vaccine and ensuring comprehensive vaccine coverage were identified as the key priorities to end the ongoing crisis by halting transmission and reducing extra intensive care demand [7]. Through the collaboration of the government, pharmaceutical companies, and academic experts, several countries have developed efficacious and clinically safe vaccines since the COVID-19 inception [8,9]. Vaccines can curb the virus spread efficiently; however, in all countries, a resistant faction of people stands against the vaccine and hinders vaccine rollout’s efficient attainment from limiting the transmission effectively [10,11]. Moreover, ensuring the maximum COVID-19 vaccine uptake in developing countries is a major challenge due to their financial and technical capacity. This negative impact of the supply-side barriers can have a multiplier effect if it is joined by the demand-side barriers. Hence the necessity of studying the demand-side factors of COVID-19 vaccine uptake intention (VUI) is necessary.

Empirical evidence suggests that vaccine uptake is crucially hindered by vaccine hesitancy [12]. Vaccine hesitancy (i.e., any delay in acceptance or refusal of vaccine despite the availability of vaccination services) has been rated among the top 10 global Public Health threats by the World Health Organization (WHO) [13,14]. Causes of vaccine hesitancy are heterogeneous and include, inter alia: religion, culture, gender, accessibility to vaccines, trust issues, and so on [14,15]. Besides vaccine hesitancy, Biasio and colleagues (2021) pointed out that vaccine literacy also can shape the VUI amid the inability to contain free-floating fake news and misinformation thanks to the uncontrolled use of the internet [16]. Additionally, eHealth literacy has been proven effective in shaping health-promoting behaviors, infection-preventive behaviors, and other types of vaccine uptake [17]. Nevertheless, the relationship of vaccine literacy and eHealth literacy with COVID-19 vaccine uptake is mostly unexplored in the LMICs [17,18,19]. Hossain and colleagues (2021) [10] once noted that most of the studies regarding vaccine uptake had been conducted in developed countries. However, a recent study looking at the vaccine acceptance in 10 LMICs across the globe has attempted to fill in the gap [20]. As Bangladesh was not among the list of the countries covered in that study, we decided to investigate what affects VUI in Bangladesh the most. For this study, we limited our interest to examine how vaccine hesitancy, eHealth literacy, and vaccine literacy have an effect on VUI.

Bangladesh launched its COVID-19 vaccination program on 27 January 2021, and by the end of October 2021, about 25% of the total population had received at least one shot of the vaccine [21]. It implies the lion’s share of the younger population is yet to receive their first jab. Young adults’ health-related behaviors, like VUI, may vary substantially from those of adults [22]. They have been identified as the low compliers of public health guidelines that involve voluntary participation [23]. The gap in research on young adults’ VUI in Bangladesh can prove costly as concerns are approaching higher than ever as the so-called “second wave” of COVID-19 has taken the highest toll on the younger people [24] compared to its previous wave. Therefore, the need to know the influencers of vaccine uptake intention of young adults is high to ensure their maximum vaccination when available to them.

To address the research gap involving young adults’ VUI in any LMIC, we aimed to explore the association of vaccine hesitancy, eHealth literacy, and vaccine literacy on VUI of young adults in Bangladesh. This study will inform the policymakers to devise the appropriate plans to maximize the vaccine uptake frequency of the young adults during and beyond the pandemic.

## 2. Materials and Methods

### 2.1. Study Setting, Design, Participants, and Sampling

Bangladesh is an LMIC according to the World Bank classification with a gross domestic product (GDP) of USD 324.339 billion and around 8.15% growth rate [25]. Young adults constitute the most significant portion of the Bangladeshi population [26]. We designed a cross-sectional study employing the Secondary and Intermediate Level Students’ Welfare Association (SILSWA) as the sampling frame. SILSWA owns a social media (Facebook) group of almost 1 million students and alumni of various educational institutions in Bangladesh. Therefore, we assumed that the Facebook group owned by SILSWA could be a rich platform containing many formally educated young adults [26]. A simple random sampling technique was applied, and Cochran’s formula, n = (z^2^pq)/d, was used to calculate the minimum required sample size for this study. Here, n equals the minimum sample size requirement, z was set at 1.96 (95% confidence level), and d implied the degree of accuracy set at 0.05. From the pilot study, 67% of participants showed the willingness to uptake the vaccine when available to them. Therefore, 0.67 was assumed as the proportion of the participants having a specific characteristic (p), and in effect, q (q = 1 – p) was equal to 0.33. The minimum required sample size for this study turned out to be 340.

We generated random numbers through the computer and invited the person with the corresponding serial number in the mentioned Facebook group’s member list via Facebook Messenger. The study’s aim, data confidentiality statement, and anonymity declarations were described in detail on the questionnaire’s first page, along with a brief description of the ongoing pandemic and the vaccination program. Upon giving consent, participants aged 18–30 years were included in the study [27]. The final sample included in the study was 343 participants.

### 2.2. Measures

#### Outcome Variable

##### VUI

VUI of the young adults was the outcome variable of this study. It was measured by the level of agreement (0 = no; 10 = definitely yes) of the participants on a single item questioning “how likely they will uptake the COVID-19 vaccine when available to them” [28].

### 2.3. Explanatory Variables of Interest

#### Vaccine Hesitancy

A 15-item instrument developed using the “5C model” of psychological antecedents was used to measure vaccine hesitancy [29]. The instrument is divided into five domains: confidence, complacency, constraints, calculation, and collective responsibility. The confidence domain contains questions regarding people’s confidence in vaccine safety. The complacency domain captures people’s complacent attitude toward the vaccine. The constraints domain relates to the psychological barriers that restrain people from getting vaccinated. The calculation domain reflects people’s cost–benefit perception toward the vaccine uptake. The collective responsibility domain captures how people think of their responsibility toward society. Each domain was individually measured with respective three items on a seven-point scale (1 = strongly disagree; 7 = strongly agree). Each domain’s mean score was computed. A higher average score indicated higher agreement of the respective domain. Vaccine hesitancy has been used as a predictor of VUI in a similar study [28]. See Appendix A for the survey questionnaires.

### 2.4. COVID-19 Vaccine Literacy

The participants’ vaccine literacy was captured by the COVID-19 vaccine literacy scale developed by Biasio and colleagues (2020) [30]. The scale was comprised of two components. The functional skills associated with vaccine literacy were measured with four items on a four-point scale (1 = often; 2 = sometimes; 3 = rarely; 4 = never). The interactive/critical skills necessary for vaccine literacy were measured with eight items on a four-point scale (1 = never; 2 = rarely; 3 = sometimes; 4 = often). The total score ranged from 16 to 48. The higher the score, the higher the COVID-19 vaccine literacy. This instrument’s Cronbach alpha was 0.86, implying excellent internal consistency [31].

### 2.5. eHealth Literacy

eHealth literacy is defined as the “ability to seek, find, understand, and appraise health information from electronic sources and apply the knowledge gained to addressing or solving a health problem” [32] Participants’ skill of looking for electronic health resources was measured with the eHealth literacy scale (eHEALS) [32]. The formal part of this instrument consists of eight items. Additionally, the scale developers have incorporated two supplementary items to measure people’s general interest in eHealth. Responses were recorded on a 5-point scale (1 = strongly disagree; 5 = strongly agree). The total score ranged from 16 to 50. A higher score meant a higher eHealth literacy. This instrument’s internal consistency was excellent, with a Cronbach alpha score of 0.90 [31].

### 2.6. Control Variables

We assumed there might be other variables which are not among our variables of interest, however, might affect the outcome. We included such control variables based on the relevant pieces of literature. We hypothesized that conspiracy theories could affect the relationship between the explanatory variables of interest and VUI [33,34]. Respondents were asked to state their beliefs about the COVID-19 related conspiracy theories, i.e., implanting a microchip in the human body with the vaccine (1 = yes; 0 = no), and the probability of being impotent after vaccination (1 = yes; 0 = no). Additionally, opinion leaders might influence the community, thereby substantially boosting the vaccine uptake intention [35]. Therefore, we included the influence of the opinion leaders as control and measured with a single-item instrument asking the participants’ level of agreement with the following statement on a five-point scale (1 = strongly disagree; 5 = strongly agree): “The COVID-19 vaccine uptake of opinion leaders (politicians, teachers, civil servants, media personnel) inspires me to uptake the vaccine”. We asked participants whether they were COVID-19 infected or not (1 = yes; 0 = no) and incorporated this into the model since young adults affected by COVID-19 were more reluctant to exhibit any preventive behavior [36]. Finally, we included fundamental demographic variables (age, sex) in the models as previous studies found a significant association between vaccine uptake intention and those demographic variables [37,38].

### 2.7. Statistical Analyses

#### 2.7.1. Descriptive Analyses

All the variables were summarized with descriptive statistics such as mean, median, and percentage of responses as appropriate. There is a debate on carrying out parametric analyses for ordinal data (for example, Likert scale data). However, Geoff Norman who is an influential medical education research methodologist, has concluded that parametric tests and statistical procedures are suitable Likert scale data and is even more robust than the nonparametric tests [39]. Pairwise correlations between vaccine uptake intention and the independent variables were computed.

#### 2.7.2. Estimation Technique

Recent pieces of literature suggest that linear regression can produce precise estimates even if the dependent variable is an ordinal one [40,41]. Kwok and colleagues [28] have successfully estimated the parameters using linear regression in their study that involves the same ordinal dependent variable used in this study. Based on this methodological and empirical evidence, our main empirical strategy involved using the Ordinary Least Squares (OLS) technique to run multiple linear regression models to capture the effect of explanatory variables of interest on VUI. OLS refers to “a model of a relationship between one or more explanatory variables and a continuous or at least interval outcome variable that minimizes the sum of square errors, where an error is the difference between the actual and the predicted value of the outcome variable” [42]. Accordingly, we estimated the following equation for each of the explanatory variables of interest:

VUI_i_ = βEVI_i_ + λX_i_ + ϵ_i_

Here, EVI is the explanatory variables of interest (5C antecedents of vaccine hesitancy, eHealth literacy, and vaccine literacy), X is the set of control variables entered into each model, and ϵ is the idiosyncratic error term. We focus on the regression coefficient β which estimates the change in VUI due to a unit change in any EVI. There was no issue of multicollinearity in any of the models as VIF values oscillated between 1 and 1.35. We reported robust standard errors for all the models to account for heteroscedasticity. For each explanatory variable of interest, we estimated three different models. Model 1 is an unadjusted model without any control variables, model 2 is a partially controlled model using individual characteristics (sex, age), and model 3 is a fully controlled model using the individual characteristics, the influence of opinion leaders and COVID-19 related experiences (have had COVID-19, COVID-19 related conspiracy theory believing). As a robustness check of the models, we considered probit specifications for each model by making VUI a dichotomous variable following the criteria (any response from 0 to 5 in the original scale equals 0 in the dichotomized scale, any response from 6 to 10 in the original scale equals 1 in the dichotomized scale) formulated by Jeong and Lee (2016) [43]. The level of significance was set at 5%. All the analyses were performed using Stata software (version 16.0).

## 3. Results

### 3.1. Descriptive Statistics

Table 1 portrays the sample characteristics and the pairwise correlation between the COVID-19 vaccine uptake intention and independent variables. The 2nd column of the table represents mean values for VUI, 5C antecedents of vaccine hesitancy, vaccine literacy, eHealth literacy, and influence of opinion leaders. This column also reports median of respondents’ age and provides response percentage for other variables. Male participants were greater in number than the female participants in the sample (58.60%). The median age of the participants was 21 years. In total, 29.45% of the participants were microchip-related conspiracy theory believers, and 15.16% believed vaccinated people might become impotent.

The mean value of the vaccine uptake intention of the sampled young adults was 7.08 with standard deviation of 3.18. The mean vaccine literacy was 33.86 (SD = 7.21) and the mean eHealth literacy was 39.39 (SD = 7.87). Vaccine confidence, collective responsibility, eHealth literacy score, age, and vaccine uptake of opinion leaders significantly and positively correlated with COVID-19 vaccine uptake intention. On the other hand, vaccine complacency, vaccine constraint, and belief that vaccinated people might become impotent significantly and negatively correlate with COVID-19 vaccine uptake intention. We did not find any significant correlation between vaccine literacy and VUI.

### 3.2. Main Analysis

Table 2 provides the results of the OLS estimations specific to each explanatory variable of interest. VUI was strongly associated with all, excluding one of the psychological antecedents of vaccine hesitancy. In all the models, people’s confidence in vaccine safety and sense of collective responsibility toward society was positively associated with VUI. The psychological constraint of getting the shots and calculating cost–benefit regarding vaccine uptake had a significant negative relationship with VUI. The full tables of the models are placed in the Supplementary Materials.

**Figure 1:**
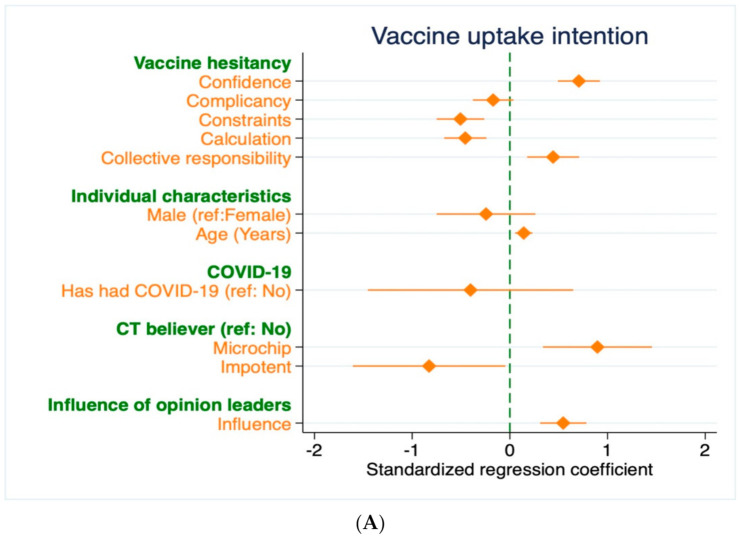

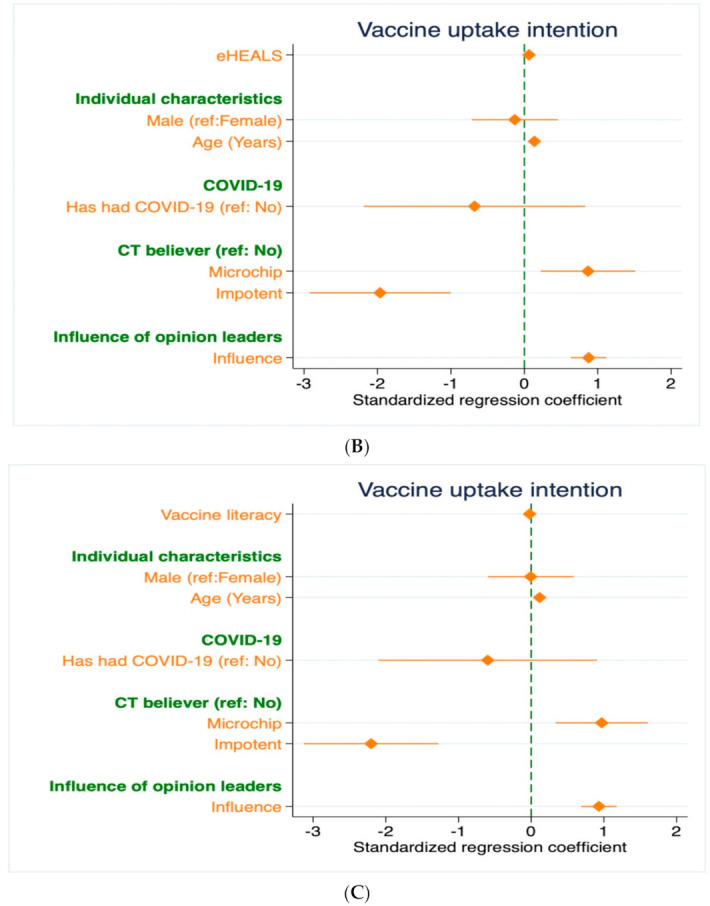
Results of OLS estimations measuring the association of explanatory variables of interest with VUI. (**A**) Results of OLS estimation measuring the association of vaccine hesitancy with VUI; (**B**) Results of OLS estimation measuring the association of eHealth literacy with VUI; (**C**) Results of OLS estimation measuring the association of vaccine literacy with VUI. Note: All the regression coefficients have been standardized to make comparable using plots.

eHealth literacy predicted the intention of vaccine uptake positively in all the models. However, vaccine literacy failed to have any influence on the VUI of young adults. Figure 1 demonstrates the plots representing estimation results from the fully controlled model for each explanatory variable. The standardized regression coefficients (variances were standardized to 1) were presented with their corresponding confidence intervals (shown by the corresponding bars). The psychological antecedents of vaccine hesitancy had more substantial effects on VUI than the eHealth literacy.

### 3.3. Robustness Checks

Table 3 shows the results of the probit models we estimated to check the robustness of our primary analyses. Our findings from the OLS estimation were reiterated by the results of the probit models except for the effect of complacency on VUI. The complacent attitude of the people negatively predicted the VUI in the partially controlled and fully controlled models. However, the respective coefficient was significant only at the 10% level in the partially controlled model.

## 4. Discussion

The study aimed to explore the effect of vaccine hesitancy, eHealth literacy, and vaccine literacy on COVID-19 VUI among young adults in Bangladesh. Confidence, constraints, calculation, and collective responsibility domains of the vaccine hesitancy emerged as the strongest predictor of VUI. Confidence about the vaccine’s safety and a sense of collective responsibility toward the society positively influence their vaccine uptake intention. However, the more they think about their cost–benefit toward vaccine uptake and the more restrictions they face to do so, the less intent they possess to have the shots. eHealth literacy shared a positive association with VUI, while vaccine literacy had no significant association.

The effects of 5C psychological antecedents measuring vaccine hesitancy like confidence, collective responsibility, calculation, and constraint on the vaccine uptake intention of young Bangladeshi adults align with a recent study conducted in China [28]. Hossain and colleagues (2021) measured the impact of 5C psychological antecedents on vaccine hesitancy of Bangladeshi people, but our study is different from that for two reasons [10]. First, we used 5C psychological antecedents to reflect the vaccine hesitancy among people following the study of Kwok and colleagues (2021) [28]. Second, the mentioned Bangladeshi study covered all ages, whereas our study focused precisely on young adults.

Young adults with more eHealth literacy had stronger intent of getting the shots, which echoes a study conducted on HPV prevention in a socioeconomically similar country [44]. To our knowledge, this study is the first of its kind to relate young adults’ eHealth literacy to COVID-19 VUI. Young adults buy the conspiracy theories running in the market like the adults in Britain and the United States, which detrimentally affects health and preventive behaviors like their vaccine uptake intention [33,45]. A person with better eHealth literacy is supposed to be more efficient in wiping out the misinformation, ill-motivated, and conspiracy theory-driven rumors and thereby ends up with a high VUI.

This study has several important theoretical and policy implications for the government before administering vaccines to young adults. First, to make young adults feel more confident about the vaccine, transmitting the latest vaccine safety updates among young adults through authentic channels is essential. Young adults in Bangladesh usually possess more information from various social media platforms regarding the COVID-19 [46,47]. Amid the misinformation flooded in social media, trust in government sources’ information is positively associated with the vaccine uptake intention [30,48]. To supplement this idea, the government can aim to enhance the eHealth literacy of young adults as an increased level of eHealth literacy will enable young adults to extract authentic health-related information more efficiently than ever. Second, possessing more civic capital will give birth to a sense of collective responsibility among young adults. To make the young adults more altruistic, inserting moral values and social norms should be started from their early education days. The altruist young adults with a better sense of collective responsibility are expected to comply with public health guidelines that require voluntary partaking, like vaccine uptake.

This study is not free from its limitations. As we used a social media group as the sampling frame, we could not explore the demographic characteristics of all the group members and therefore, the outcomes in this study might not be representative of all the young adults in Bangladesh. Since the responses are digitally recorded, we could not control for the environmental biases, for example, that arose from the housing conditions of the participants. Questions on VUI, vaccine literacy, eHealth literacy, and vaccine hesitancy might generate a tendency to mask the participants’ original intention, knowledge, and attitude through socially desirable answers. In this study, we were unable to control for this social desirability bias, and the outcomes might contain the impacts of this bias. We only found the association between the vaccine uptake intention and its predictors, but the causal relation is yet to be explored. Future studies should keep all the limitations of this study in consideration and focus more on finding the causal impacts of the predictors on VUI using a larger sample size than that used in this study.

## Figures and Tables

**Table 1:** Sample characteristics and bivariate correlation with COVID-19 vaccine uptake intention.

Variable	Mean/N (%)	SD	r
Vaccine uptake intention	7.08	3.18	
Vaccine hesitancy			
Confidence	4.58	1.66	0.47 ***
Complacency	3.4	1.45	−0.15 ***
Constraints	2.84	1.4	−0.37 ***
Calculation	5.77	1.45	−0.06
Collective responsibility	4.47	1.04	0.26 ***
Vaccine literacy	33.86	7.21	−0.05
eHealth literacy	39.39	7.87	0.24 ***
Age	Median = 21	Range = 12	0.12 **
Influence of opinion leaders	3.36	1.34	0.46 ***
Sex of the respondents			−0.05
Female	142 (41.40%)		
Male	201 (58.60%)		
COVID-19 patient			−0.04
No	324 (94.46%)		
Yes	19 (5.54%)		
Conspiracy theory believer (microchip)			0.07
No	242 (70.55%)		
Yes	101 (29.45%)		
Conspiracy theory believer (impotent)			−0.3 ***
No	291 (84.84%)		
Yes	52 (15.16%)		

Notes: *** *p* < 0.01; ** *p* < 0.05; r = correlation coefficient; SD = standard deviation. This table provides the characteristics of the sample included in this study. Mean values were reported for VUI, all domains of vaccine hesitancy, vaccine literacy, eHealth literacy, and influence of opinion leaders. The median value was reported for age. All other variables have been summarized using percentages. This table also provides a bivariate correlation of all the explanatory variables with VUI.

**Table 2:** Ordinary least squares (OLS) estimation of effects of vaccine hesitancy, eHealth literacy, and vaccine literacy on VUI.

Variable	Model 1	Model 2	Model 3
	Effect of Vaccine Hesitancy on VUI		
Vaccine hesitancy			
Confidence	0.94 ***	0.98 ***	0.71 ***
	(0.10)	(0.10)	(0.11)
Complacency	−0.11	−0.11	−0.16
	(0.11)	(0.11)	(0.11)
Constraints	−0.53 ***	−0.53 ***	−0.52 ***
	(0.13)	(0.13)	(0.12)
Calculation	−0.62 ***	−0.54 ***	−0.45 ***
	(0.11)	(0.11)	(0.11)
Collective responsibility	0.67 ***	0.58 ***	0.46 ***
	(0.14)	(0.14)	(0.13)
Constant	5.24 ***	2.31	1.17
	(1.00)	(1.45)	(1.42)
Controls	Null	Partial	Full
Observations	343	343	343
VIF	1.33	1.30	1.31
R-squared	0.40	0.42	0.49
	Effect of eHealth literacy on VUI		
eHealth literacy	0.10 ***	0.11 ***	0.07 ***
	(0.02)	(0.02)	(0.02)
Constant	3.18 ***	−0.50	−1.52
	(0.85)	(1.46)	(1.32)
Controls	Null	Partial	Full
Observations	343	343	343
VIF	-	1.02	1.09
R-squared	0.06	0.09	0.31
	Effect of vaccine literacy on VUI		
Vaccine literacy	−0.02	−0.02	−0.02
	(0.02)	(0.02)	(0.02)
Constant	7.79 ***	5.28 ***	2.07
	(0.78)	(1.48)	(1.38)
Controls	Null	Partial	Full
Observations	343	343	343
VIF	-	1.00	1.06
R-squared	0.00	0.02	0.29

Notes: This table provides results of the OLS models estimating the association of vaccine hesitancy, eHealth literacy, and vaccine literacy with VUI. The controls included in each model specification are None (no controls included) (model 1), Partial controls (model 2), and Full controls (model 3). Partial controls include individual-level characteristics (sex and age). Full controls include partial controls_ plus COVID-19 related experiences (have had COVID-19, conspiracy theory believing) and influence of opinion leaders. Robust standard errors have been reported in the parentheses. *** *p* < 0.01.

**Table 3:** Probit estimation of effects of vaccine hesitancy, eHealth literacy, and vaccine literacy on VUI.

Variable	Model 1	Model 2	Model 3
	Effect of Vaccine Hesitancy on VUI		
Vaccine hesitancy			
Confidence	0.43 ***	0.47 ***	0.38 ***
	(0.07)	(0.07)	(0.07)
Complacency	−0.10	−0.12 *	−0.16 **
	(0.07)	(0.07)	(0.07)
Constraints	−0.23 ***	−0.23 ***	−0.24 ***
	(0.07)	(0.07)	(0.07)
Calculation	−0.31 ***	−0.27 ***	−0.25 ***
	(0.07)	(0.07)	(0.08)
Collective responsibility	0.31 ***	0.26 ***	0.22 **
	(0.09)	(0.09)	(0.10)
Constant	0.02	−1.86 **	−3.08 ***
	(0.60)	(0.93)	(1.00)
Controls	Null	Partial	Full
Observations	343	343	343
	Effect of eHealth literacy on VUI		
eHealth literacy	0.04 ***	0.05 ***	0.04 ***
	(0.01)	(0.01)	(0.01)
Constant	−1.10 ***	−2.87 ***	−4.05 ***
	(0.36)	(0.70)	(0.78)
Controls	Null	Partial	Full
Observations	343	343	343
	Effect of vaccine literacy on VUI		
Vaccine literacy	−0.01	−0.01	−0.01
	(0.01)	(0.01)	(0.01)
Constant	0.78 **	−0.31	−1.96 ***
	(0.34)	(0.62)	(0.72)
Controls	Null	Partial	Full
Observations	343	343	343

Notes: This table provides results of the probit models estimating the association of vaccine hesitancy, eHealth literacy, and vaccine literacy with VUI. The controls included in each model specification are None (no controls included), Partial controls, and Full controls. Partial controls include individual-level characteristics (sex and age). Full controls include partial controls_ plus COVID-19 related experiences (have had COVID-19, conspiracy theory believing) and influence of opinion leaders. Robust standard errors have been reported in the parentheses. *** *p* < 0.01, ** *p* < 0.05, * *p* < 0.1.

## Data Availability

The raw data collected in this study are available on request from the corresponding author (S.D.N.).

## Supplementary Materials

The following are available online at https://www.mdpi.com/article/10.3390/vaccines9121405/s1, Table S1: OLS estimation measuring the impact of vaccine hesitancy on VUI, Table S2: Probit estimation measuring the impact of vaccine hesitancy on VUI, Table S3: OLS estimation measuring the impact of eHealth literacy on VUI, Table S4: Probit estimation measuring the impact of eHealth literacy on VUI, Table S5: OLS estimation measuring the impact of vaccine literacy on VUI, Table S6: Probit estimation measuring the impact of vaccine literacy on VUI.

## Appendix A

1. Vaccine uptake intention (Kwok et al., 2020):

How likely they will take the COVID-19 vaccine when available to them on a 11-point Likert scale (1 = definitely no; 10 = definitely yes; rate your intention by any number between 1 and 10).

* Changed the scale to make it consistent with other scales as all of them start from 1.

2. Vaccine hesitancy (5C scale) (Betsch et al., 2018): (7 points scale: 1 = Strongly Disagree; 7 = Strongly Agree; rate your hesitancy by any number between 1 and 10)

Domains: Confidence (1–3; Alpha = 0.84), Complacency (4–6; Alpha = 0.57), Constraints (6–9; Alpha = 0.61); Calculation (10–12; Alpha = 0.63), Collective responsibility (13–15; Alpha = 0.50)

**Items**
1. I am completely confident that vaccines are safe.
2. Vaccinations are effective.
3. Regarding vaccines, I am confident that public authorities decide in the best interest of the community.
4. Vaccination is unnecessary because vaccine-preventable diseases are not common anymore.
5. My immune system is so strong, it also protects me against diseases.
6. Vaccine-preventable diseases are not so severe that I should get vaccinated.
7. Everyday stress prevents me from getting vaccinated.
8. For me, it is inconvenient to receive vaccinations.
9. Visiting the doctor’s makes me feel uncomfortable; this keeps me from getting vaccinated.
10. When I think about getting vaccinated, I weigh benefits and risks to make the best decision possible.
11. For each and every vaccination, I closely consider whether it is useful for me.
12. It is important for me to fully understand the topic of vaccination, before I get vaccinated.
13. When everyone is vaccinated, I don’t have to get vaccinated, too. (R)
14. I get vaccinated because I can also protect people with a weaker immune system.
15. Vaccination is a collective action to prevent the spread of diseases.
- R means reverse scale

3. Vaccine Literacy Scale (Biasio et al., 2020):

When reading or listening to information about future COVID-19 vaccines or current vaccines (4 points scale: 1 = often, 2 = sometimes, 3 = rarely, 4 = never)

(1) Did you find words you didn’t know?
(2) Did you find that the texts were difficult to understand?
(3) Did you need much time to understand them?
(4) Did you or would you need someone to help you understand them?

When looking for information about future COVID-19 vaccines or current vaccines (4 points scale: 1 = often, 2 = sometimes, 3 = rarely, 4 = never):

(1) Have you consulted more than one source of information?
(2) Did you find the information you were looking for?
(3) Have you had the opportunity to use the information?
(4) Did you discuss what you understood about vaccinations with your doctor or other people?
(5) Did you consider whether the information collected was about your condition?
(6) Have you considered the credibility of the sources?
(7) Did you check whether the information was correct?
(8) Did you find any useful information to make a decision on whether or not to get vaccinated?

4. eHealth Literacy Scale (Norman and Skinner, 2006)

1. How useful do you feel the Internet is in helping you in making decisions about your health? (5 points scale: 1 = Not useful at all; 5 = Very useful)
2. How important is it for you to be able to access health resources on the Internet? (5 points scale: 1 = Not important at all; 5 = Very important)
3. I know what health resources are available on the Internet (5 points scale: 1 = Strongly disagree; 5 = Strongly agree)
4. I know where to find helpful health resources on the Internet (5 points scale: 1 = Strongly disagree; 5 = Strongly agree)
5. I know how to find helpful health resources on the Internet (5 points scale: 1 = Strongly disagree; 5 = Strongly agree)
6. I know how to use the Internet to answer my questions about health (5 points scale: 1 = Strongly disagree; 5 = Strongly agree)
7. I know how to use the health information I find on the Internet to help me (5 points scale: 1 = Strongly disagree; 5 = Strongly agree)
8. I have the skills I need to evaluate the health resources I find on the Internet (5 points scale: 1 = Strongly disagree; 5 = Strongly agree)
9. I can tell high quality health resources from low quality health resources on the Internet (5 points scale: 1 = Strongly disagree; 5 = Strongly agree)
10. I feel confident in using information from the Internet to make health decisions (5 points scale: 1 = Strongly disagree; 5 = Strongly agree)

* Note: Questions #1 and #2 are recommended as supplementary items for use with the eHEALS to understand consumer’s interest in using eHealth in general. These items are not a formal part of the eHealth Literacy scale, which comprises questions #3–10.
