# Supplementary material for: Role of Vaccine Hesitancy, eHealth Literacy, and Vaccine Literacy in Young Adults’ COVID-19 Vaccine Uptake Intention in a Lower-Middle-Income Country
Source: Vaccines (Basel). 2021 Nov 29;9(12):1405. doi: 10.3390/vaccines9121405 (PMC8704098; doi:10.3390/vaccines9121405)
Supplement: Supplementary file 1 [file vaccines-09-01405-s001.zip › vaccines-1415814-supplementary.pdf]

Supplementary Materials:

Table S1: OLS estimation measuring the impact of vaccine hesitancy on VUI

OLS estimation measuring impact of vaccine hesitancy on vaccine uptake intention

	(1)	(2)	(3)
VARIABLES	VUI	VUI	VUI
Vaccine hesitancy			
Confidence	0.94***	0.98***	0.71***
	(0.10)	(0.10)	(0.11)
Complacency	-0.11	-0.11	-0.16
	(0.11)	(0.11)	(0.11)
Constraints	-0.53***	-0.53***	-0.52***
	(0.13)	(0.13)	(0.12)
Calculation	-0.62***	-0.54***	-0.45***
	(0.11)	(0.11)	(0.11)
Collective responsibility	0.67***	0.58***	0.46***
	(0.14)	(0.14)	(0.13)
Sex (ref: Female)			
Male		-0.44	-0.26
		(0.27)	(0.26)
Age (in years)		0.14***	0.15***
		(0.05)	(0.05)
COVID-19 affected (ref: No)			
Yes			-0.39
			(0.53)
Conspiracy theory believer (ref: No)			
Microchip implantation			0.91***
			(0.28)
Risk of being impotent			-0.86**
			(0.40)
Vaccine-maker country (ref: UK)			
India			0.40
			(0.25)
Opinion leaders			0.56***
			(0.12)
Constant	5.24***	2.31	1.17
	(1.00)	(1.45)	(1.42)
Observations	343	343	343
R-squared	0.40	0.42	0.49

Robust standard errors in parentheses

*** $p<0.01$, ** $p<0.05$, * $p<0.1$

Table S2: Probit estimation measuring the impact of vaccine hesitancy on VUI

Probit model estimating impact of vaccine hesitancy on vaccine uptake intention

	(1)	(2)	(3)
VARIABLES	VUI	VUI	VUI
Vaccine hesitancy			
Confidence	0.43***	0.47***	0.38***
	(0.07)	(0.07)	(0.07)
Complacency	-0.10	-0.12*	-0.16**
	(0.07)	(0.07)	(0.07)
Constraints	-0.23***	-0.23***	-0.24***
	(0.07)	(0.07)	(0.07)
Calculation	-0.31***	-0.27***	-0.25***
	(0.07)	(0.07)	(0.08)
Collective responsibility	0.31***	0.26***	0.22**
	(0.09)	(0.09)	(0.10)
Sex (ref: Female)			
Male		-0.15	-0.03
		(0.17)	(0.17)
Age (in years)		0.09***	0.11***
		(0.03)	(0.03)
COVID-19 affected (ref: No)			
Yes			0.40
			(0.44)
Conspiracy theory believer (ref: No)			
Microchip implantation			0.61***
			(0.23)
Risk of being impotent			-0.43
			(0.26)
Vaccine-maker country (ref: UK)			
India			0.35**
			(0.17)
Opinion leaders			0.30***
			(0.07)
Constant	0.02	-1.86**	-3.08***
	(0.60)	(0.93)	(1.00)
Observations	343	343	343

Robust standard errors in parentheses
*** p<0.01, ** p<0.05, * p<0.1

Table S3: OLS estimation measuring the impact of eHEALS on VUI

OLS estimation measuring impact of e-Heals on vaccine uptake intention

VARIABLES	(1)<br>VUI	(2)<br>VUI	(3)<br>VUI
eHEALS	0.10***	0.11***	0.07***
	(0.02)	(0.02)	(0.02)
Sex (ref: Female)			
Male		-0.50	-0.14
		(0.33)	(0.30)
Age (in years)		0.16***	0.14***
		(0.05)	(0.05)
COVID-19 affected (ref: No)			
Yes			-0.67
			(0.77)
Conspiracy theory believer (ref: No)			
Microchip implantation			0.88***
			(0.33)
Risk of being impotent			-1.99***
			(0.49)
Vaccine-maker country (ref: UK)			
India			0.27
			(0.29)
Opinion leaders			0.89***
			(0.13)
Constant	3.18***	-0.50	-1.52
	(0.85)	(1.46)	(1.32)
Observations	343	343	343
R-squared	0.06	0.09	0.31

Robust standard errors in parentheses
*** p<0.01, ** p<0.05, * p<0.1

Table S4: Probit estimation measuring the impact of eHEALS on VUI

Probit model estimating impact of e-Heals on vaccine uptake intention

VARIABLES	(1)<br>VUI	(2)<br>VUI	(3)<br>VUI
eHEALS	0.04***	0.05***	0.04***
	(0.01)	(0.01)	(0.01)
Sex (ref: Female)			
Male		-0.15	0.01
		(0.15)	(0.16)
Age (in years)		0.07***	0.08***
		(0.02)	(0.03)
COVID-19 affected (ref: No)			
Yes			0.16
			(0.39)
Conspiracy theory believer (ref: No)			
Microchip implantation			0.46**
			(0.20)
Risk of being impotent			-0.84***
			(0.25)
Vaccine-maker country (ref: UK)			
India			0.25
			(0.16)
Opinion leaders			0.37***
			(0.06)
Constant	-1.10***	-2.87***	-4.05***
	(0.36)	(0.70)	(0.78)
Observations	343	343	343

Robust standard errors in parentheses

*** p<0.01, ** p<0.05, * p<0.1

Table S5: OLS estimation measuring the impact of vaccine literacy on VUI

OLS estimation measuring impact of vaccine literacy on vaccine uptake intention

VARIABLES	(1)<br>VUI	(2)<br>VUI	(3)<br>VUI
Vaccine literacy	-0.02	-0.02	-0.02
	(0.02)	(0.02)	(0.02)
Sex (ref: Female)			
Male		-0.36	-0.01
		(0.34)	(0.30)
Age (in years)		0.12**	0.12***
		(0.05)	(0.05)
COVID-19 affected (ref: No)			
Yes			-0.59
			(0.77)
Conspiracy theory believer (ref: No)			
Microchip implantation			0.98***
			(0.32)
Risk of being impotent			-2.22***
			(0.47)
Vaccine-maker country (ref: UK)			
India			0.23
			(0.30)
Opinion leaders			0.94***
			(0.13)
Constant	7.79***	5.28***	2.07
	(0.78)	(1.48)	(1.38)
Observations	343	343	343
R-squared	0.00	0.02	0.29

Robust standard errors in parentheses
*** p<0.01, ** p<0.05, * p<0.1

Table S6: Probit estimation measuring the impact of vaccine literacy on VUI

Probit model estimating impact of vaccine literacy on vaccine uptake intention

VARIABLES	(1)<br>VUI	(2)<br>VUI	(3)<br>VUI
Vaccine literacy	-0.01	-0.01	-0.01
	(0.01)	(0.01)	(0.01)
Sex (ref: Female)			
Male		-0.09	0.07
		(0.14)	(0.16)
Age (in years)		0.06**	0.07***
		(0.02)	(0.02)
COVID-19 affected (ref: No)			
Yes			0.18
			(0.39)
Conspiracy theory believer (ref: No)			
Microchip implantation			0.51***
			(0.19)
Risk of being impotent			-0.96***
			(0.24)
Vaccine-maker country (ref: UK)			
India			0.23
			(0.16)
Opinion leaders			0.39***
			(0.06)
Constant	0.78**	-0.31	-1.96***
	(0.34)	(0.62)	(0.72)
Observations	343	343	343

Robust standard errors in parentheses
*** p<0.01, ** p<0.05, * p<0.1
